# Association of Postoperative Ileus With Length of Hospital Stay in Abdominal Surgery Patients

**DOI:** 10.7759/cureus.97393

**Published:** 2025-11-20

**Authors:** Syed Taqi Askari Shah, Aakif Yousaf, Rashid Minhas, Muhammad Idrees Shabbir, Abdullah Khan, Yasmin Sajid

**Affiliations:** 1 Trauma and Orthopedics, Queen's Medical Centre, Nottingham University Hospitals NHS Trust, Nottingham, GBR; 2 Surgery, Sahiwal Teaching Hospital, Sahiwal, PAK; 3 Surgery, Sir Ganga Ram Hospital, Fatima Jinnah Medical University, Lahore, PAK; 4 General and Laparoscopic Surgery, Aga Khan Medical Centre, Gilgit, PAK; 5 General Surgery, Gujranwala Medical Complex, Gujranwala, PAK

**Keywords:** abdominal surgery, hospital stay, ileum, postoperative ileus, surgery

## Abstract

Background and aim: Postoperative ileus (POI) is a frequent complication following abdominal surgeries, characterized by delayed gastrointestinal motility and associated with increased patient morbidity and healthcare burden. This study aimed to determine the association between postoperative ileus and length of hospital stay (LOS) among patients undergoing abdominal surgery.

Methods: This retrospective observational study was conducted at District Headquarters (DHQ) Teaching Hospital, Gujranwala, Pakistan, from January 2024 to December 2024. A total of 335 patients were included in this study. Data were retrieved from patients’ hospital files, surgical records, and discharge summaries. Collected variables included demographic details (age and gender), clinical characteristics (comorbidities such as diabetes and hypertension), surgical details (type of surgery, approach, and duration), postoperative opioid usage, development of POI, and total length of hospital stay.

Results: POI occurred in 94 patients (28.1%). The mean LOS was significantly longer in the POI group (9.6±3.2 days) compared to those without POI (5.4±2.1 days; p<0.001). Open surgery, emergency procedures, operative time >120 min, and opioid use were significantly associated with POI. In logistic regression, POI independently predicted prolonged hospital stay (>7 days) with an adjusted odds ratio of 4.82 (95% CI: 2.91-7.98, p<0.001).

Conclusion: Postoperative ileus significantly prolongs hospital stay in patients undergoing abdominal surgery. Identifying high-risk patients and implementing preventive strategies such as minimizing opioid use, promoting early mobilization, and adopting minimally invasive techniques can reduce POI incidence and enhance postoperative recovery.

## Introduction

Postoperative ileus (POI) is a reversible dysfunction of the coordinated bowel motility that often results after abdominal surgeries [1]. Although postoperative ileus is initially a normal physiological reaction to surgical trauma, it becomes a pathological condition when it persists beyond the expected recovery period, leading to significant adverse effects on patient outcomes. Clinically, it presents with abdominal distension, pain, delayed passage of flatus and stool, nausea, vomiting, and an inability to tolerate oral intake [2]. Such symptoms have an adverse effect on recovery and complicate the discharge, and therefore, POI is envisioned as a core aspect of postoperative management of all patients, particularly abdominal surgery patients. POI in different individuals and surgical procedures differs in duration, but usually gets resolved in two to three days [3,4]. Patients continue to have an elevated risk of complications (e.g., fluid and electrolyte imbalances, malnutrition, aspiration pneumonia, and wound dehiscence because of vomiting or straining) when symptoms become protracted after this window. The impact of POI goes beyond the discomfort of the patient; it is also a leading cause of excessive length of hospital stay (LOS) due to hospital readmissions, and high expenditures used on medical care [5,6].

There are various pathophysiological processes that support POI. Surgical disturbance of the intestines triggers an inflammatory reaction of a local nature with the participation of resident macrophages and the secretion of systemically active pro-inflammatory cytokines, including interleukin-6 (IL-6) and tumor necrosis factor-alpha (TNF-α) [7]. This destroys the usual enteric neural communication, resulting in inhibition of smooth muscle contractions. Moreover, administration of perioperative opioids constitutes the second risk factor that would increase POI by inhibiting gastrointestinal motility through the stimulation of µ-opioid receptors [8]. Additional factors are the depth and the duration of surgery, surgical fluid changes, alterations in the electrolyte system, and immobility after surgery. The kind of abdominal surgery is also important in the occurrence of POI. What is very risky as compared to the less invasive usage of laparoscopes is the open surgeries, especially those that involve the colon, the small intestine, or the retroperitoneal surgeries [9,10]. Nevertheless, total prevention of this complication, even with laparoscopic interventions, does not exist, particularly when extensive bowel mobilization is performed. An age greater than 65 years, male gender, already existing comorbidities (e.g., diabetes, hypothyroidism), nutritional deficiency states, and drugs all predispose patients towards the development of POI [11]. Over the past years, a number of measures have been used to curb the occurrence and impact of POI. These include early mobilization, early oral feeding, opioid avoidance or minimization through multimodal analgesia, and the use of prokinetic agents or peripheral receptor antagonists (e.g., alvimopan). The systematic approach to surgery suggested by Enhanced Recovery After Surgery (ERAS) protocols suggests an evidence-based, holistic policy of perioperative care and has proved to diminish POI and decrease length of hospital stay (LOS) across various types of surgery [12]. Regardless of these innovations, POI remains a substantial postoperative complication, especially in a resource-limited environment in which the implementation of the core ERAS principles can be uneven. Hospital stay is one of the essential parameters of the postoperative improvement and healthcare productivity. Such prolongation of LOS is unnecessary not only because of the direct cost-related developments to healthcare institutions but also because of the possible side effects, such as the patients facing the risk of contracting hospital-acquired infections, venous thromboembolism, and medication-related side effects, as well as the emotional distress [13]. LOS is also associated with bed turnover rates and surgical throughput in most healthcare systems, and thus, such complications as POI directly affect the institutional productivity and resource distribution. Evidence-based literature in high-income nations has been conducted regarding the association of POI and extended LOS, but low- and middle-income countries have yet to be explored. The importance of this knowledge gap is peculiar due to the existing difficulties in these regions, including delays in presentation, poor uniformity of perioperative care, heterogeneous rates of access to minimally invasive surgery, and a lack of availability of specialized recovery pathways such as ERAS [14].

This study aimed to assess the association between postoperative ileus (POI) and the length of hospital stay in patients undergoing abdominal surgery and to identify major risk factors, such as surgical approach, operative duration, and opioid use, that contribute to its development.

## Materials and methods

This was a retrospective observational study conducted at District Headquarters (DHQ) Teaching Hospital, Gujranwala, Pakistan, from January 2024 to December 2024. A total of 335 patients were included in the study. Non-probability consecutive sampling was used to recruit eligible patients. Patients aged 18 years and above who had undergone elective or emergency abdominal surgery, including gastrointestinal, hepatobiliary, and gynecologic procedures, were included in this study. Additionally, only those with complete medical records and postoperative follow-up of at least five days were included. Patients with pre-existing conditions, such as bowel obstruction or paralytic ileus, were excluded. Also, individuals with incomplete or missing data were excluded from the study, as well as those who expired within 48 hours postoperatively.

Data collection procedure

Data were retrieved from patients’ hospital files, surgical records, and discharge summaries. Collected variables included demographic details (age and gender), clinical characteristics (comorbidities such as diabetes and hypertension), surgical details (type of surgery, approach, and duration), postoperative opioid usage, development of POI, and total length of hospital stay. Each patient’s data were coded and anonymized before analysis. Postoperative ileus was defined as the persistence of at least two of the following features beyond postoperative day three: abdominal distension, absence of passage of flatus or stool, nausea or vomiting, inability to tolerate oral intake, or radiological evidence of bowel dilatation without mechanical obstruction. This definition was adapted from existing standardized clinical guidelines. The primary outcome variable was the length of hospital stay (in days), calculated from the date of surgery to the date of discharge. It was analyzed both as a continuous variable and as a binary variable (≤7 days vs. >7 days) to identify predictors of prolonged hospitalization. Secondary outcomes included the overall incidence of POI in the study population, as well as its association with specific surgical variables, such as operative duration, surgical approach (open vs. laparoscopic), and type of surgery (elective vs. emergency).

Data analysis

Data were entered and analyzed using SPSS version 26 (Armonk, NY: IBM Corp.). Continuous variables were presented as mean±standard deviation (SD) or median with interquartile range (IQR), depending on data distribution. Categorical variables were summarized using frequencies and percentages. The independent samples t-test was used to compare LOS between patients with and without POI. Chi-square test was applied for categorical comparisons. A p-value less than 0.05 was considered statistically significant.

## Results

This study included 335 surgical patients with a mean age of 48.2±15.3 years, and 57.6% (n=193) were male. Most surgeries were elective (62.1%, n=208) and 71.9% (n=241) were performed using an open surgical approach. Gastrointestinal resections were the most common procedure type (35.5%, n=119), followed by cholecystectomies (26.2%, n=88) and gynecologic surgeries (18.5%, n=62), reflecting a diverse operative cohort skewed toward abdominal and digestive surgeries (Table 1).

**Table 1:** Baseline characteristics of patients (n=335).

Variables	Total (n=335)
Age (years), mean±SD	48.2±15.3
Gender (male), n (%)	193 (57.6)
Type of surgery (elective), n (%)	208 (62.1)
Surgical approach (open), n (%)	241 (71.9)
Gastrointestinal resections, n (%)	119 (35.5)
Cholecystectomies, n (%)	88 (26.2)
Gynecologic procedures, n (%)	62 (18.5)

Out of 335 patients, 94 (28.1%) developed postoperative ileus (POI). These patients had significantly longer hospital stays, averaging 9.6±3.2 days compared to 5.4±2.1 days in the no-POI group (p<0.001). Prolonged hospitalization (>7 days) occurred in 69.1% of POI patients vs. 19.5% without POI (p<0.001). POI patients more frequently underwent open surgery (87.2% vs. 65.9%, p<0.001), emergency surgery (38.3% vs. 21.6%, p=0.002), and had longer operative times (138.4±42.7 min vs. 96.3±30.2 min, p<0.001). Opioid use was also significantly higher in the POI group (78.7% vs. 49.4%, p<0.001), underscoring its role as a modifiable risk factor (Table 2).

**Table 2:** Comparison of outcomes in patients with and without postoperative ileus. Chi-square test for categorical comparisons (e.g., open surgery, emergency surgery, opioid use). Independent samples t-test for continuous variables (e.g., length of hospital stay and operative time). POI: postoperative ileus

Variables	POI (n=94)	No POI (n=241)	p-Value	Test statistic
Length of hospital stay (days), mean±SD	9.6±3.2	5.4±2.1	<0.001	t=12.02
Hospital stay >7 days, n (%)	65 (69.1)	47 (19.5)	<0.001	Chi-square=28.09
Open surgery, n (%)	82 (87.2)	159 (65.9)	<0.001	Chi-square=28.09
Emergency surgery, n (%)	36 (38.3)	52 (21.6)	0.002	Chi-square=9.74
Operative time (min), mean±SD	138.4±42.7	96.3±30.2	<0.001	t=9.93
Opioid use, n (%)	74 (78.7)	119 (49.4)	<0.001	Chi-square=34.52

Multivariable logistic regression identified postoperative ileus as the strongest independent predictor of prolonged hospital stay (>7 days), with an adjusted odds ratio (AOR) of 4.82 (95% CI: 2.91-7.98, p<0.001). Open surgery was also significantly associated (AOR: 2.35, 95% CI: 1.42-3.91, p<0.001), as was operative time over 120 min (AOR: 1.89, 95% CI: 1.13-3.16, p=0.015). These findings suggest that POI, surgical invasiveness, and procedural duration are major contributors to extended recovery (Table 3).

**Table 3:** Multivariable logistic regression for predictors of prolonged hospital stay (>7 days).

Predictors	Adjusted odds ratio (AOR)	95% Confidence interval	p-Value
Postoperative ileus	4.82	2.91-7.98	<0.001
Open surgery	2.35	1.42-3.91	<0.001
Operative time >120 min	1.89	1.13-3.16	0.015

Gastrointestinal resections had the highest rate of POI, with 48 out of 119 patients (40.3%) affected, showing strong statistical significance (p<0.001). Other procedures, such as cholecystectomies (15.9%), gynecologic abdominal surgeries (17.7%), and appendectomies (17.1%) had lower POI rates. Notably, “other” surgeries (e.g., urologic or hepatic) had a high POI rate of 48.4%, although no specific p-value was reported for these subgroups. These results suggest GI and complex abdominal procedures carry a higher POI risk (Table 4).

**Table 4:** Frequency of postoperative ileus by surgical type. Chi-square test for comparing the frequencies of POI cases across different types of surgery. Chi-square test value=24.87, df=4, p<0.001. POI: postoperative ileus

Type of surgery	Total patients (n)	POI cases	No POI cases	p-Value
Gastrointestinal resections, n (%)	119	48 (40.3)	71 (59.7)	<0.001
Cholecystectomies, n (%)	88	14 (15.9)	74 (84.1)	-
Gynecologic abdominal surgeries, n (%)	62	11 (17.7)	51 (82.3)	-
Appendectomies, n (%)	35	6 (17.1)	29 (82.9)	-
Other (e.g., urologic, hepatic), n (%)	31	15 (48.4)	16 (51.6)	-

Length of stay was longer in the POI group: 69.1% (n=65) stayed >7 days compared to just 19.5% (n=47) in the non-POI group. Only 9.6% of POI patients were discharged within five days vs. 58.5% of non-POI patients. The overall mean stay was 9.6±3.2 days for POI and 5.4±2.1 days for non-POI, with a statistically significant difference (p<0.001, Mann-Whitney U test). This reinforces the impact of POI on hospital resource utilization and patient recovery (Table 5).

**Table 5:** Length of stay distribution in patients with and without POI. Mann-Whitney U test for comparing the distribution of length of stay between POI and non-POI groups. The chi-square test was used to assess the difference in categorical outcomes (e.g., length of stay categories). The statistical value for the chi-square test for the comparison of hospital stay categories (≤5, 6-7, >7 days) was chi-square=132.13 (p<0.001) for the total group comparison, and the Mann-Whitney U test statistic for the comparison of mean lengths of stay was U=5786.5 (p<0.001). POI: postoperative ileus

Length of stay (days)	POI group (n=94), n (%)	No POI group (n=241), n (%)	Total (n=335), n (%)	p-Value	Test statistic
≤5 Days	9 (9.6)	141 (58.5)	150 (44.8)	<0.001	Chi-square=132.13
6-7 Days	20 (21.3)	53 (22.0)	73 (21.8)	<0.001	-
>7 Days	65 (69.1)	47 (19.5)	112 (33.4)	<0.001	Chi-square=132.13
Mean±SD	9.6±3.2	5.4±2.1	6.7±3.1	-	U=5786.5

## Discussion

In the present study, the relationship between length of hospital stay (LOS) and postoperative ileus (POI) was explored in patients admitted with abdominal surgery. Here, POI was a major cause of unnecessarily prolonged hospital stay because total in-hospital length of stay was almost twice as long in patients with POI as in those without POI (9.6±3.2 vs. 5.4±2.1 days, p<0.001). In this study, postoperative ileus (POI) is defined as the persistence of at least two of the following features beyond postoperative day three: abdominal distension, absence of passage of flatus or stool, nausea or vomiting, inability to tolerate oral intake, or radiological evidence of bowel dilatation without mechanical obstruction. Besides, POI turned out to be an independent factor contributing to long-term hospitalization in multivariate analysis, which also supports its clinical and logistical significance during postoperative care. The incidence of POI in our study population amounted to 28.1%, similar to the findings of prior literature in the range of 10-30% depending on the type of surgery and population-specific characteristics [15]. The hypothesis that gastrointestinal motility was impeded by higher volume of bowel manipulation, surgical stress, and other inflammatory components is supported by its dramatically higher risk in open and emergency procedures [16]. This supports the evidence in Enhanced Recovery After Surgery (ERAS) procedures, where the focus is on minimally invasive procedures and early mobilization to prevent ileus. The fact that the use of opioids in our data is strongly associated with POI (78.7% in POI group vs. 49.4% in non-POI group, p<0.001) reports a well-known, modifiable risk factor. When administered, opioids bind to the µ-opioid receptors located in the gastrointestinal tract and stimulate the inhibition of the peristalsis process and aggravate ileus. These outcomes contribute to the increasing movement towards multimodal analgesia and opioid-sparing methods with regard to perioperative care [17]. Use of non-opioid pain medications, regional blocks, and early nutrition can treat POI by implementing faster recovery along with a decrease in its prevalence.

The other significant factor was the operating time, with the POI patients accounting for a significantly longer surgery (138.4±42.7 vs. 96.3±30.2 min, p<0.001). The long procedure might indicate more complicated cases or increased exposure of the bowels during surgery, both of which have been known to cause ileus. This is an indication that the surgical team ought to be efficient and contemplate policies, such as laparoscopic surgical techniques, where feasible, to reduce the time and decrease invasiveness. POI was highly linked to the type of surgery as well. The most frequent one (40.3%) was gastrointestinal resections, in contrast to some of the other procedures, such as cholecystectomy (15.9%) or gynecologic procedures (17.7%). This finding is biologically plausible, as intestinal resections are extensive procedures that often involve significant bowel manipulation and disruption of the enteric nervous system (ENS) pathways. Notably, 69.1% of patients with POI experienced prolonged hospital stays compared to only 19.5% of those without POI (p<0.001). Such outcomes have major implications for healthcare systems, especially in low- and middle-income countries like Pakistan, where efficient resource utilization, bed turnover, and management of hospital congestion are ongoing challenges.

This is a potential benefit that anyone may have, because lowering POI rates is not just a way of making patients improve better, but may streamline institutional performance and affordability. We also found evidence in the results connected to the idea that POI is a potential target of the perioperative quality-improvement intervention. Prophylaxis interventions, which may comprise gum chewing, early ambulation, intravenous fluid management, and medications (e.g., alvimopan), might be adopted in postoperative practices. Also, patient education and nurse attention to the early manifestations of ileus may result in early intervention and avoid the possibility of developing clinically severe manifestations. Our results are consistent with the data published in high-income contexts and support the view that POI is of concern on a global scale regarding healthcare systems. Another study conducted reviewed that POI increased LOS by five days on average, which could explain this disparity found in our study design [18]. On the same note, Courtney et al. showed open surgery and the use of opioids as the most important risk factors, consistent with our multivariate analysis [19]. The uniformity in worldwide writings confirms the strength of POI as a quantifiable and adjustable indication of postoperative results.

This study has several limitations. One limitation of the study is its retrospective design, which relies on existing medical records and discharge summaries, potentially leading to missing or incomplete data. Additionally, the study was conducted at a single center, limiting the generalizability of the findings to other healthcare settings or populations. The use of non-probability consecutive sampling may have introduced selection bias, as it does not ensure a random representation of the broader patient population. Furthermore, the study did not account for potential confounders, such as the severity of comorbidities or variations in postoperative care protocols, which could have influenced the development of postoperative ileus and the length of hospital stay.

## Conclusions

It is concluded that postoperative ileus is a common and clinically significant complication following abdominal surgery, strongly associated with prolonged hospital stay. Patients who developed POI had nearly double the duration of hospitalization compared to those without it, highlighting its substantial impact on recovery and healthcare resource utilization. Key predictors of POI included open surgical approach, emergency procedures, prolonged operative time, and perioperative opioid use, all of which are potentially modifiable through evidence-based perioperative practices.

## References

[1] Animaw FC, Asresie MB, Endeshaw AS (2025). Postoperative ileus and associated factors in patients following major abdominal surgery in Ethiopia: a prospective cohort study. BMC Surg.

[2] Vather R, Josephson R, Jaung R, Robertson J, Bissett I (2015). Development of a risk stratification system for the occurrence of prolonged postoperative ileus after colorectal surgery: a prospective risk factor analysis. Surgery.

[3] Venara A, Neunlist M, Slim K, Barbieux J, Colas PA, Hamy A, Meurette G (2016). Postoperative ileus: pathophysiology, incidence, and prevention. J Visc Surg.

[4] Traeger L, Koullouros M, Bedrikovetski S, Kroon HM, Moore JW, Sammour T (2023). Global cost of postoperative ileus following abdominal surgery: meta-analysis. BJS Open.

[5] Senagore AJ (2007). Pathogenesis and clinical and economic consequences of postoperative ileus. Am J Health Syst Pharm.

[6] Zhu Z, He B, He J (2025). Preoperative malnutrition is a risk factor for prolonged postoperative ileus for patients undergoing gastrointestinal surgery. Front Nutr.

[7] Li ZL, Zhao BC, Deng WT, Zhuang PP, Liu WF, Li C, Liu KX (2020). Incidence and risk factors of postoperative ileus after hysterectomy for benign indications. Int J Colorectal Dis.

[8] Lee TH, Lee JS, Hong SJ (2015). Risk factors for postoperative ileus following orthopedic surgery: the role of chronic constipation. J Neurogastroenterol Motil.

[9] Sever K, Ozbek C, Goktas B, Bas S, Ugurlucan M, Mansuroglu D (2014). Gastrointestinal complications after open heart surgery: incidence and determinants of risk factors. Angiology.

[10] Svatek RS, Fisher MB, Williams MB (2010). Age and body mass index are independent risk factors for the development of postoperative paralytic ileus after radical cystectomy. Urology.

[11] Watkins EL, Schellack N, Abraham V, Bebington B (2021). Men and those with a history of smoking are associated with the development of postoperative ileus following elective colorectal cancer resection at a private academic hospital in Johannesburg, South Africa: a retrospective cohort study. Front Surg.

[12] Namba Y, Hirata Y, Mukai S (2021). Clinical indicators for the incidence of postoperative ileus after elective surgery for colorectal cancer. BMC Surg.

[13] Mbuthia FC (2020). Incidence and risk factors of post-operative ileus in adult patients at Kenyatta national hospital. https://erepository.uonbi.ac.ke/handle/11295/154061.

[14] Sueta MA, Golden N, Prawira MD (2022). Risk factors for post-operative ileus: a retrospective study in tertiary referral hospital in Indonesia. Open Access Maced J Med Sci.

[15] van Bree SH, Bemelman WA, Hollmann MW (2014). Identification of clinical outcome measures for recovery of gastrointestinal motility in postoperative ileus. Ann Surg.

[16] Moghadamyeghaneh Z, Hwang GS, Hanna MH (2016). Risk factors for prolonged ileus following colon surgery. Surg Endosc.

[17] Vather R, Trivedi S, Bissett I (2013). Defining postoperative ileus: results of a systematic review and global survey. J Gastrointest Surg.

[18] Armellini A, Chew S, Johnston S, Muralidharan V, Nikfarjam M, Weinberg L (2024). The hospital costs of complications following major abdominal surgery: a retrospective cohort study. BMC Res Notes.

[19] Courtney A, Clymo J, Dorudi Y, Moonesinghe SR, Dorudi S (2024). Scoping review: the terminology used to describe major abdominal surgical procedures. World J Surg.

